# Diazotrophic growth of free-living *Rhizobium etli:* Community-like metabolic modeling of growing and non-growing nitrogen-fixing cells

**DOI:** 10.1371/journal.pone.0325888

**Published:** 2025-06-27

**Authors:** Maryam Afarin, Fereshteh Naeimpoor

**Affiliations:** 1 Biotechnology Research Laboratory, School of Chemical, Petroleum and Gas Engineering, Iran University of Science and Technology, Tehran, Iran; Federal University Dutse, NIGERIA

## Abstract

*Rhizobium etli*, a nitrogen-fixing bacterium, grows both in symbiosis (with plants) and in free-living state. While most metabolic models focus on its symbiotic form, this study refined the existing *i*OR363 model to account for free-living growth. By addition of a biomass formation reaction followed by model curation growth was simulated using various N-sources (NH₃, NO₂, and NO₃). At fixed succinate uptake rate (4.16 mmol/gDWC/h), ammonia yielded the highest growth rate of 0.259 h ⁻ ¹. To represent free-living N-fixing *R. etli*, a novel two-member community-like model, consisting of both growing and differentiated non-growing N-fixing cells with ammonia exchange, was developed. The XFBA approach, based on community Flux Balance Analysis (cFBA), was formulated to maintain fixed abundances rather than assuming equal growth rates. With a non-growing:growing abundance ratio of 1:9 in community, N-fixation resulted in lower growth rate of 0.1933 h ⁻ ¹ due to the high energy demand of N₂ assimilation compared to ammonia. Sensitivity analysis revealed that increased abundance of N-fixing cells from 5 to 30% led to decreases of 10% in N_2_-fixation and 25% in growth rate of growing member. Furthermore, Principal Component Analysis identified oxidative phosphorylation, TCA cycle, and glycolysis as key pathways differentiating flux distributions across N-sources. At high uptake of oxygen, causing nitrogenase inactivity, cytochrome *bd* oxidase was activated to scavenge oxygen, though at the cost of lower growth rate (by 12% per mmol increase in O_2_ uptake/gDWC/h). This study provided a platform to obtain insights to free-living state of *R. etli* which may have applications for other diazotrophs.

## Introduction

Biological dinitrogen (N_2_) fixation by diazotrophic microorganisms (some cyanobacteria, bacteria and archaea), either in free-living state or in symbiotic relationship with other organisms, has played a pivotal role in providing bio-accessible forms of nitrogenous compounds (e.g., ammonia) in biosphere (aquatic and agricultural environments) [1]. Prokaryotes ranging from obligate anaerobe to obligate aerobes, can fix nitrogen by the activity of nitrogenase enzyme requiring 16 moles of ATP per one mole of N_2_, which is a substantial energy demand and contributes to the low growth rates of nitrogen-fixing microorganisms [2].

While aerobic metabolism promotes generation of ATP necessary for nitrogen fixation, it has been shown that nitrogenase enzymes, due to their metal cofactors, are oxygen-sensitive, posing a paradox [3]. To address this, aerobic nitrogen-fixers have developed mechanisms to protect nitrogenase from oxygen damage. Creating or choosing environments with minimal internal oxygen is one of the strategies used for protection of nitrogenase. Filamentous cyanobacteria form specialized non-growing N-fixing cells called heterocysts with thick cell walls protecting the enzyme alongside the growing cells utilizing the fixed nitrogen. Some other bacteria can form symbiotic relationship with plants, residing in the root nodules where oxygen concentration is low [4]. Diazotrophic bacteria can also secrete extracellular polymeric substances limiting oxygen penetration [2,5]. Some photosynthetic nitrogen fixers grow when exposed to light (during the day) while nitrogen fixation occurs during the dark period (night), where photosynthesis is inactive and hence oxygen is not produced [4].

“Respiratory protection” is another strategy to protect nitrogenase which can be employed by free-living diazotrophs (in contrast to symbionts of a host) or those lacking the previously mentioned strategies. The bacterial respiratory chain is highly flexible and modular, featuring a diverse array of dehydrogenases, quinone electron carriers, cytochrome *bc1* complex, as well as various quinol oxidases and reductase complexes. Two important oxidases catalyzing the final stage of respiratory chain, where O₂ is reduced to H₂O, are cytochrome *c* and cytochrome *bd* oxidases, each of which is utilized under specific circumstances. Equations 1 and 2 represent the reactions of cytochrome *bd* and cytochrome *c* oxidases, respectively [6]. It should be noted that cytochrome *c* acts as an electron carrier, while cytochrome *bd* oxidase does not participate directly in the proton pumping process, and quinol (QH₂) is oxidized to quinone (Q) in the reaction.


$$\begin{array}{c} 2\;QH_{2}+O_{2}+2\;H_{in}+\to 2\;Q^{ox}+2\;H_{2}O+2\;H_{out}+ \end{array}$$ (1)

$$\begin{array}{cc} & \;4\;\text{cytochrome}\;c\;\text{(reduced)}+{\text{O}}_{2}+8\;{\text{H}}_{\text{in}}+\to \;4\;\text{cytochrome}\;c\;\text{(oxidized)}\\ & +2H_{2}O+4\;H_{out}+ \end{array}$$ (2)

$$\begin{array}{c} ADP+2\;H_{out}++pi\to \;ATP+H_{2}O+H_{in}+ \end{array}$$ (3)

Cytochrome *c*, with a lower affinity for oxygen activates under aerobic conditions, while when bacteria need to use oxygen-sensitive enzymes such as nitrogenase, cytochrome *bd* can play a role in scavenging O₂ to protect these types of enzymes, showing the inducibility of respiratory chain in bacteria [6,7]. Furthermore, cytochrome *c* pumps 4 protons per transferred electron (Eq. 2), whereas cytochrome *bd* pumps 2 protons (Eq. 1). This results in higher ATP generation (Eq. 3) by cytochrome *c* per oxygen consumption. Conversely, higher oxygen consumption per ATP generation can be achieved by cytochrome *bd* [2,6], which in turn lowers oxygen level in the culture, making the environment favorable for nitrogenase activity. Additionally, evidences have shown increased expression of cytochrome *bd* in N-fixing bacteria at elevated levels of oxygen. This increase supports the role of cytochrome *bd* in reducing intracellular oxygen levels and hence protecting the nitrogenase enzyme [2,3,7].

Rhizobia, one important genus of diazotrophs, have recently been the focus of extensive research. These bacteria are gram-negative α-proteobacteria, categorized as strict aerobes, and include genera such as *Azorhizobium*, *Bradyrhizobium*, *Mesorhizobium*, *Rhizobium*, and *Sinorhizobium.* Among them, *Rhizobium* is the most prevalent which exhibits remarkable metabolic flexibility. They can utilize various carbon sources (e.g., glucose via glycolysis and malate, fumarate, or succinate via the TCA cycle) and diverse nitrogen sources such as ammonium, nitrite, and nitrate. In the absence of fixed nitrogen sources, they fix atmospheric nitrogen (N₂). While primarily known as symbiotic bacteria of plant roots, *Rhizobium* can also grow in a free-living form, maintaining their diazotrophic behavior in both states. Additionally, *Rhizobium* is capable of accumulating intercellular substances such as poly-β-hydroxybutyrate (PHB) under both symbiotic and free-living conditions. This accumulation, which occurs under low oxygen concentrations, serves to balance the reducing equivalents like NAD(P)H generated during carbon metabolism [8–12].

Since nitrogen fixation was originally observed in the non-growing *Rhizobium* bacteroids (differentiated bacterial cells within root nodules of leguminous plants), it was believed that exclusively non-growing cells can fix nitrogen. This notion however was challenged by further observations that free-living *Rhizobium* fixes nitrogen while growing [8,9,12–14].

To address this issue, Ludwig based on experimental practice hypothesized that free-living *Rhizobium* cultures consist of mixed population of non-growing N-fixing and growing cells [8]. In nitrogen-deficient liquid suspension cultures, N-fixing cells provide ammonium, which is then utilized by the growing cells. This hypothesis suggests prokaryotic differentiation, where metabolically specialized, terminally non-growing cells function cooperatively in a higher cellular order. In fact, this differentiation occurs to protect the nitrogenase enzyme from oxygen damage, similar to the formation of heterocysts in filamentous cyanobacteria as previously mentioned [8].

To better comprehend the cellular behavior under various conditions, without the costs associated with experimental practices, flux balance analysis (FBA) methodology has been widely used over the past two decades [15–19]. This methodology allows estimation of flux distribution within cells using biochemical reaction network of microorganisms alongside performing mass balances on intracellular metabolites under pseudo steady state condition.

FBA has been applied to single species of Rhizobia to investigate nitrogen fixation by reconstructing various metabolic models, namely *i*OR363, *i*OR450, *i*HZ565 and *i*CC541 [20–23]. These models lacked a conventional biomass formation reaction since single non-growing cells was considered in a symbiotic state with a host (excluded in modelling). Their objective function primarily focused on the production of symbiotic metabolites exchanged with the host, such as ammonium and amino acids, and the accumulation of substances like glycogen and PHB. Two models, *i*YY1101 [24] and *i*AQY970 [25], have been developed for free-living growth of *Bradyrhizobium diazoefficiens* and *Sinorhizobium fredii*, respectively, both species of *Rhizobia*, and include biomass reactions. However, *i*YY1101 disregards nitrogen fixation and uses nitrate as the nitrogen source, *i*AQY970 notably includes both growth and nitrogen fixation. However, in the latter, the fixed ammonia is modeled as leaving the system without contributing to biomass synthesis, which may limit its ability to capture nitrogen assimilation under free-living diazotrophic conditions. Altogether, all previous models have either focused on nitrogen fixation in a symbiotic state or on a free-living non-diazotrophic form. These models are not applicable for capturing the complexity of *Rhizobium* population in its free-living state, where segregated growing and non-growing N-fixing cells coexist within a community.

Several algorithms have so far been developed to analyze metabolic models of microbial communities. FBA for community [26] and OptCom [27] consider the members of a community as compartments, among which interactions could take place. Growth of nitrogen fixing filamentous cyanobacteria using two-cell types (heterocyst and growing cells) was previously modeled using super-compartments [28–30]. Although compartmentalization provides a framework for analysis of communities, it ignores the relative abundances of members. This issue was further addressed in cFBA [31] and SteadyCom [18] algorithms. In addition to employing the abundances of members, these two algorithms assume equal growth rates of all members in the community. A new framework is therefore necessary for analyzing free-living growth of *Rhizobium* with the coexistence of both growing and non-growing N-fixing members in the community.

In this work, the behavior of *Rhizobium etli*, a member of the *Rhizobium* genera with the unique ability to thrive both as a free-living bacterium and in symbiotic relationships with plant roots and microalgae [32], was for the first time investigated in free-living state using a community-like model. Simulations were carried out by designing an algorithm termed XFBA which extends the traditional FBA formulation by incorporating member abundances similar to cFBA, though avoiding the balanced growth rate assumption of cFBA. This approach allowed greater flexibility in choosing different objective functions matching the reality.

## Methods

### Metabolic models

#### Source model of *R. etli.*

To reconstruct a community-like metabolic model of *R. etli* population consisting of both growing and non-growing N-fixing members, the first reconstructed single-species model of this genus (*i*OR363), consisting of 387 reactions and 371 metabolites was used [20]. It was chosen due to its relatively small network, allowing detailed examination and curation of reactions to develop robust and reliable metabolic model. This model, originally constructed to simulate the symbiotic non-growing state of *R. etli*, lacked biomass formation reaction and an artificial reaction (a linear combination of some amino acids, storage materials and ammonium) was considered as its objective function. To create the two variants of growing and non-growing N-fixing cells, this model was curated as follows.

#### Model of growing member.

Curation of *i*OR363 model was carried out by incorporation of a biomass formation reaction to provide a model suitable for growing member. Due to the lack of such reaction for *R. etli* in literature, the biomass formation reaction set taken from the metabolic model of *B. diazoefficiens* [24] belonging to the same genus (Rhizobia) was used, assuming similar biomass composition. Assuming *B. diazoefficiens* cells consisting of seven macromolecules (protein: 50.1, DNA: 3, RNA: 8.5, phospholipids: 0.9, peptidoglycan: 2.5, lipopolysaccharides: 3.4, capsular and extracellular polysaccharides: 15.7 and poly-β-hydroxybutyrate (PHB): 15.9 in w/w %), biomass formation reaction was formulated by considering the mmol of monomers making 1 gram of biomass.

**Fig 1:**
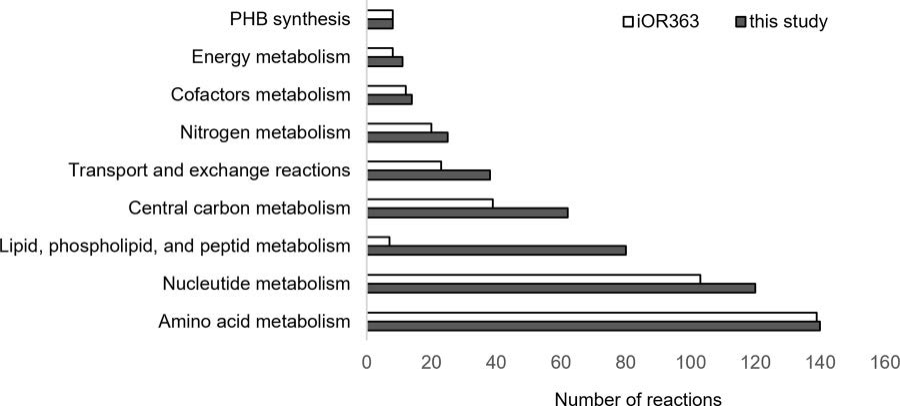
Comparison of metabolic reaction distribution in R. etli models: iOR363 vs. the curated model developed in this study.

Addition of this reaction to the source model however caused numerous gaps which were meticulously identified and cured by addition of all necessary biosynthetic reactions of monomers/precursors as well as phospholipids, peptidoglycan, lipopolysaccharides, and polysaccharides. Gaps were primarily flagged by gap detection algorithms which led to some model curations, however, manual curations were essential as these algorithms could not identify the gap for metabolites involved in reversible reactions. These metabolites were hence connected to the reaction network by addition of necessary reactions from databases such as KEGG and Biocyc. This effort culminated in a refined model comprising 496 reactions and 457 metabolites after insertion of 108 reactions. Fig 1 compares the number of reactions categorized into nine key metabolic pathways between the *i*OR363 model and the curated model named *i*OR363GM (iOR363 Growing Model) developed in this study. The curated model (*i*OR363GM) is available as S1 file in supplementary material.

#### Model of nitrogen-fixing non-Growing member.

**Fig 2:**
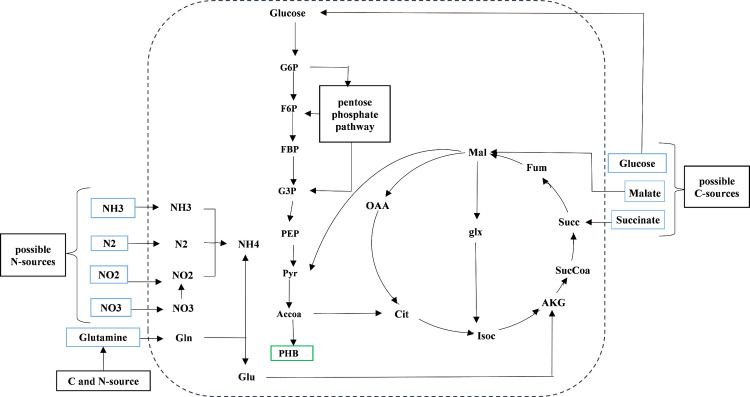
Metabolic map of the R. etli model. Blue boxes represent alternative carbon and nitrogen sources. The full names of the abbreviated intracellular metabolites are provided in metabolite list in the S1 file of supplementary material.

The model of non-growing member of *R. etli* population was created by omission of biomass formation reaction from the refined model of growing member. To consider extra production of PHB in non-growing cells [24], an exchange flux was considered for PHB in this model. Fig 2 shows metabolic map of *R. etli* model including important pathways.

#### Model of cooperative population of *R. etli.*

The two single-species models, refined for non-growing and growing members, were used to construct a community metabolic model consisting of two *R. etli* members: non-growing nitrogen fixer and growing cell utilizing the ammonium secreted by the first member. This community-like model was created using “createMultipleSpeciesModel” function of COBRA toolbox, which generates a compartmentalized community model, allowing the members to share metabolites within a common space.

**Fig 3:**
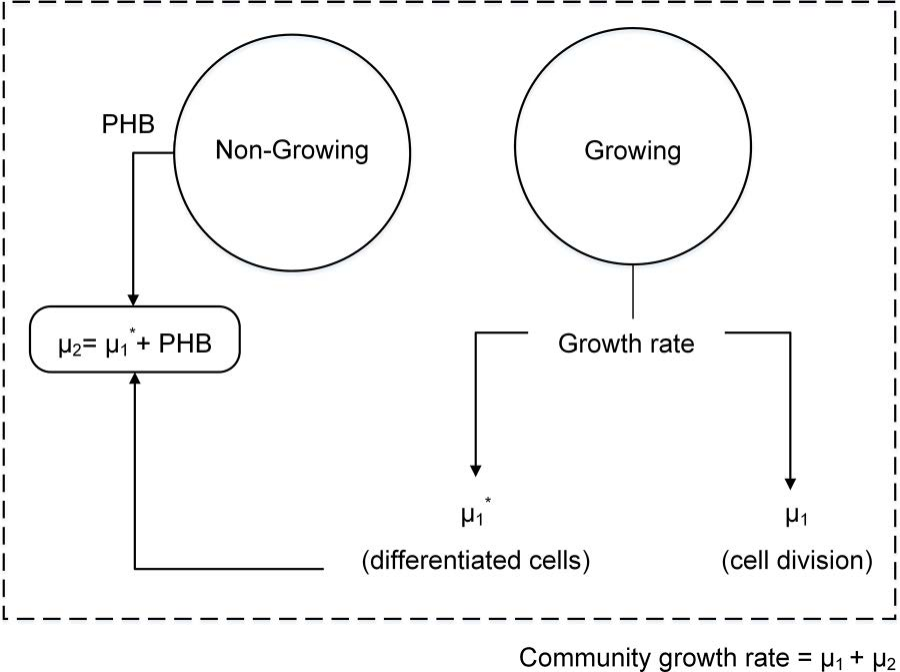
Schematic representation of free living R. etli: growing cells undergo cell division, with a portion differentiating into non-growing, nitrogen-fixing cells.


In our community model, the abundances of growing and non-growing cells were set to 0.9 and 0.1, respectively, to reflect the reality. This was based on observation of 1 non-growing N-fixing cell for every 9 growing cells in the real arrangement of filamentous N-fixing cyanobacteria [33]. To keep this abundance over time, some growing cells should differentiate to non-growing. This was modeled by defining two distinct biomass formation reactions for the growing member: one accounting for cell growth (*µ*_*1*_) and the other for growing cells to be differentiated to non-growing cells (*µ*_1_*). A schematic of community model is given in Fig 3. Differentiated cells were supposed to be added to the existing non-growing cells to provide constant abundances when growing cells increase. Another point to be considered in the model was the higher PHB content of non-growing cells of *R. etli* (50%) compared to that of growing cells (15.9%) [34]. To account for extra PHB biosynthesis by non-growing member, the model was supplemented with an exchange flux for PHB (*v*_PHB, non-growing_), the value of which being proportional to differentiated cells (*µ*_1_*) as shown in Eq. 4:

$$v_{PHB,non-growing}(\frac{mmol}{gDW.h})=\alpha \times \mu_{1}^{*}$$ (4)

with *α* = 7.9303 accounting for the extra PHB needed to increase PHB content of non-growing cells to 50%. PHB production by non-growing cell alongside the differentiated cells contribute to the increase in non-growing cells (*µ*_*2*_). To have constant abundances of members, Eq. 5 should be satisfied.


$$\mu_{2}=\mu_{1}^{*}+\frac{MW_{PHB}\times v_{PHB,non-growing}}{1000}=\frac{1}{9}(\mu_{1})$$ (5)

Using Eqs. 4 and 5, *µ*_1_^*^ was obtained as a linear function of *µ*_*1*_ as given in Eq. 6. This constraint ensures both constant abundances of members and the higher PHB content of non-growing cells.


$$\mu_{1}^{*}=\frac{1}{9}\frac{1}{1+\frac{\alpha \times MW_{PHB}}{1000}}\mu_{1}$$ (6)

The growth rate of the growing cells (*µ*₁**) was used as the objective function to maximize. This choice also leads to the maximization of the total community growth rate (*µ*₁** + *µ*₂**), since the growth rate of the non-growing N-fixing cells was constrained to be a fixed fraction of *µ*₁**, reflecting their constant relative abundance. Therefore, maximizing µ₁ inherently results in a proportional increase in *µ₂*, effectively maximizing the total community growth rate.

Furthermore, nitrogen fixation was not selected as the modeling objective. Given that the model represents the free-living state of *R. etli*, where there is no plant host, the primary biological goal is population expansion rather than nitrogen export. Therefore, maximizing the growth rate of the growing cells provides a biologically meaningful and computationally sufficient objective.

The community model named *i*OR363FL (*i*OR363 Free Living) consists of 1,012 reactions and 833 metabolites is available as S2 file in supplementary material. Maintenance energy (ATPM) was considered for both non-growing and growing cells to account for energy requirements unrelated to growth. Due to the lack of organism-specific data for *R. etli*, we assumed that its energy consumption is comparable to that of the closely related species *B. diazoefficiens*. Accordingly, ATP maintenance (ATPM) values of 1 and 2 mmol/gDWC/h (millimoles per gram dry weight of the community per hour) were assigned to the growing and N-fixing cells, respectively, based on values previously reported for *B. diazoefficiens* [24]. This choice is supported by the phylogenetic similarity between the two organisms and reflects common practice in metabolic modeling when direct measurements are unavailable. The higher ATPM value for the non-growing, N-fixing cells was assumed due to their larger cell size and potentially higher maintenance demands.

Similarly, the biomass composition used in this study was adapted from *B. diazoefficiens* due to the absence of a species-specific biomass reaction for *R. etli*. While this represents an approximation, it is supported by studies showing that predictions of genome-scale models is not highly sensitive to the stoichiometry of biomass components [35,36]. Moreover, even when species-specific biomass data is available, it often includes inherent experimental uncertainties and highly depends on growth condition. Therefore, it has been demonstrated that using biomass compositions from closely related species can yield reliable and biologically meaningful simulation results, particularly when the overall metabolic context is preserved

### XFBA formulation

Simulating the behavior of a community-like *R. etli* population utilizing the compartmentalized framework led to the creation of a unified stoichiometric matrix (S), which represents the reaction networks of the community members along with a shared compartment. This matrix (S) can be utilized in various analytical methods, such as flux balance analysis (FBA). However, as previously mentioned, FBA does not inherently account for population distribution within the community. While this limitation has been addressed in methods like cFBA and SteadyCom, these approaches assume equal growth rates for all community members, making them unsuitable for modeling a community-like *R. etli* population where one member has a zero growth rate.

To overcome this limitation, XFBA was developed based on FBA and cFBA. XFBA allows consideration of species abundances in community while excludes the equal growth rates of members. Additionally, this method offers greater flexibility in defining the objective function, whereas cFBA and SteadyCom consider the community growth rate as objective function.

To formulate XFBA for a two-member community, the stoichiometric matrix (S) must be multiplied by a diagonal matrix (X) composed of three diagonal sub-matrices to perform mass balances on metabolites using aggregate fluxes. The diagonal elements of the first two matrices are the abundance of that member (X_member_). The third matrix is an identity matrix, with its dimension matching the number of exchange reactions in the shared compartment. This operation modifies the stoichiometric matrix such that S_x _= S × X, where S_x_ replaces the original S matrix in the FBA formulation. It should be mentioned that by using fixed abundances of members, the problem remains linear and can be solved by linear solvers. Furthermore, abundances of members can be varied to investigate their effect on behavior of community.

Also, as the growth rates of members are not equal in XFBA, their abundances would not remain constant over time. To address this, a constraint was added to XFBA, requiring that the growth rates of the members be related by a coefficient such as that calculated for our community (see Eq. 5). More generally, this relationship is expressed in Eq. 7.


$$\mu_{2}=\frac{abundanceofmember2(\;\%)}{abundanceofmember1(\;\%)}(\mu 1)$$ (7)

The constraint in Eq. 7 in XFBA ensures constant abundances over time without assuming equal growth rates of members.

### Computational experiments

To explore the metabolic behavior of *R. etli* under varying conditions, computational experiments were designed to simulate growth on different nitrogen and carbon sources, as well as under N-fixing condition. The first set of experiments focused on comparing the effects of carbon sources (succinate, malate, and glucose) on growth rate and carbon utilization efficiency. The second set examined the impact of N-sources (ammonia, nitrite, nitrate, and molecular nitrogen) on growth and metabolic fluxes. Additionally, the effect of metabolite exchange between growing and non-growing members in the community model was analyzed to assess its impact on community growth rate. Also, sensitivity of modeling results to abundances investigated. Simulations explored the activation of cytochrome *bd* in high oxygen uptake, aiming to investigate its role in balancing oxygen detoxification and energy production. Finally, Principal Component Analysis (PCA) was performed to identify differences in flux distributions with various nitrogen sources.

## Results and discussion

### Growth of *R. etli* on common nitrogen and carbon sources

Growth of free-living *R. etli* has been previously investigated experimentally using various N-sources such as ammonia and nitrate as well as different C-sources such as succinate, malate, and glutamine [11,32,37]. Additionally, N-free medium has been exploited to examine growth of this bacterium when fixing molecular nitrogen [8].

In this study, the impact of different sources of carbon (succinate, malate, and glucose) and nitrogen (ammonia, nitrite, and nitrate) on growth of *R. etli* was separately investigated by applying FBA methodology. Using the developed metabolic model of growing *R. etli, i*OR363GM, six computational experiments were conducted where uptake of various C-sources were allowed one at a time alongside ammonia as the sole N-source in runs 1, 2, and 3 (see Table 1), while the effect of different N-sources was examined using succinate, the most commonly used substrate in experimental studies of *R. etli*, as the sole C-source in runs 4 and 5 (see Table 1). Additionally, the effect of glutamine containing both C- and N-atoms was assessed in run 6. To make the computational results more realistic, specific succinate uptake rate was set to 4.16 mmol/gDWC/h. This value was estimated using the experimental data [11] leading to an experimental growth rate of 0.2125 h ⁻ ¹. Uptake fluxes of other C-sources were then adjusted to provide the same flux of C-atom as succinate. Calculated specific rates of growth, uptake of N-source as well as products formation for these computational experiments are illustrated and compared in Table 1.

**Table 1:** Effect of various C- and N-sources on growth and selected fluxes of R. etli, all rates in mmol/gDWC/h.

Run no.	Subject of study	C- and N-sources				µ (h^-1^)	CO_2_ (NH_3_) release rate
		Sources		Uptake rates			
		C*	N	C**	N		
1	C-sources	Glc	NH_3_	2.773	2.758	0.348	2.165 (0)
2		Mal		4.16	1.938	0.245	6.468 (0)
3		Suc		4.16	2.052	0.259	5.868 (0)
4	N-sources	Suc	NO_2_	4.16	1.599	0.202	8.243 (0)
5			NO_3_		1.468	0.185	8.936 (0)
6	C- & N-sources	Gln		3.328		0.275	5.213 (4.48)

* Suc: succinate, Mal: malate, Glc: glucose, Gln: glutamine.

** Fixed rates of C-source uptake.

Among the examined C-sources, glucose resulted in the highest rates of growth (0.348 h ⁻ ¹) and ammonia uptake (2.758 mmol/gDWC/h). This showed that glucose efficiently supported cell growth, leading to the lowest CO_2_ emission (2.165 mmol/gDWC/h). Growth on succinate (0.259 h ⁻ ¹) was only slightly higher than that on malate (0.245 h ⁻ ¹) and about 26% lower than that on glucose. Comparison of growth rate on succinate (at 4.16 mmol/gDWC/h) and ammonia with the experimentally obtained value of 0.2125 h ⁻ ¹ show about 18% lower value for the latter. This can be justified by the fact that we targeted the optimal growth rate while in reality cells may have other or a combination of targets.

Considering that the same C-flux was set for the three C-sources, different growth rates can be attributed to the varying oxidation energy of C-atom in their structure. Both succinate and malate are four C-atom intermediates of TCA cycle while their oxidation states are different. Succinate can be oxidized to malate by releasing one mole of FADH_2_, as reducing power. Therefore, C-atoms of malate, which are more oxidized than those in succinate, have lower oxidation capacity, leading to lower growth rate. In contrast, C-atoms of glucose being less oxidized compared to those in both succinate and malate can result in much higher growth rates. Taking the identical C-fluxes into account, lower CO_2_ emissions corresponded to higher growth rates.

Examination on the effect of different N-sources showed that ammonia, nitrite (NO_2_) and nitrate (NO_3_) resulted in the highest to lowest growth rates in presence of succinate as C-source, while CO_2_ emission was in the reverse order. These findings highlighted ammonia as the most favorable N-source. This can be explained by the fact that N-incorporation into cell materials succeeds through ammonia, glutamate and glutamine. Since biosynthesis of the latter two N-compounds from oxo-glutarate (an intermediate of TCA cycle) also requires ammonia, any other N-source should be transformed to ammonia before entering the N-metabolism. Ammonia is the most reduced form of N-atom, while nitrite and nitrate are partially and completely oxidized form of N-atoms, respectively. These oxidized N-sources should hence be reduced at the expense of reducing powers before incorporation into cell components. A part of C-source therefore oxidizes to carbon dioxide to provide the necessary reducing powers and this in turn lowers the available C-source for cell growth. Having a higher oxidation state, nitrate resulted in lower growth and higher CO_2_ formation as compared to nitrite.

Glutamine, serving as both C- and N-sources, resulted in higher growth compared to most substrates, except glucose. In this case, glutamine was catabolized into glutamate and further to oxo-glutarate alongside ammonia. By joining TCA cycle, oxo-glutarate supported growth as C-source while ammonia severed as N-source. Due to the imbalance between C- and N-sources, a part of ammonia (4.48 mmol/gDWC/h) was excreted into the extracellular space. Glutamine-grown cells have previously been reported to be C-starved and N-sufficient [32] which aligns with our results showing the excretion of excess ammonia. CO₂ emission (5.213 mmol/gDWC/h) in case of glutamine as substrate (see Table 1) was comparable to succinate and malate but higher than glucose, indicating less efficient carbon utilization relative to glucose.

These results underscore the importance of substrate selection in optimizing *R. etli* growth and productivity. While ammonia and glucose emerge as the most efficient N- and C-sources respectively, the metabolic behavior of glutamine shows more flexibility.

### Growth and nitrogen fixation in community-like population of *R. etli*

One of the key metabolic capabilities of *R. etli* is the fixation of atmospheric nitrogen (N₂), which enables its cultivation in N-source free conditions, thereby reducing the costs. In this study, a community model was reconstructed (*i*OR363FL) to simulate the differentiation of a portion of the *R. etli* population into N-fixing non-growing cells, while the rest of population could grow on the fixed nitrogen by the non-growing cells, making a two member community. XFBA algorithm was developed to simulate the behavior of this community-like population. Specific information on the abundances of the two members was required for application of XFBA, however, no data was available for *R. etli*. Therefore, data was taken from the similar N-fixation of filamentous cyanobacteria with heterocyst differentiation [28]. Accordingly, population abundances were set to 0.1 for the N-fixing member and 0.9 for the growing member. Nonetheless, the assumed abundances were further varied to assess the sensitivity of results.

**Fig 4:**
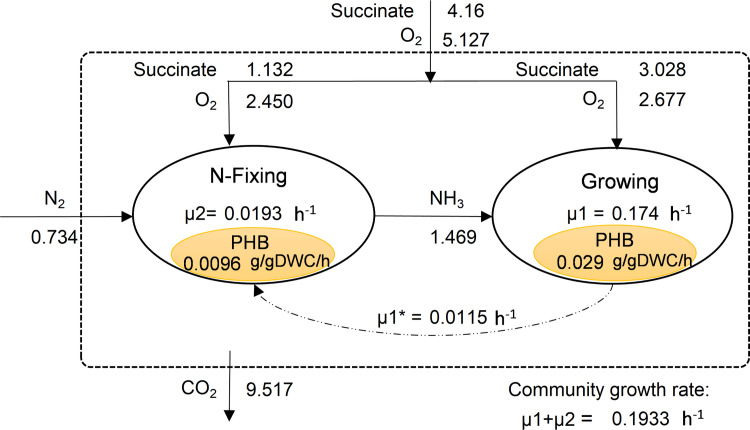
Flux distribution in N₂ fixing community of R. etli with ammonia as the sole exchanged metabolite between members. Numbers shown next to arrows show flux values in mmol/gDWC/h.

Fig 4 illustrates the flux distribution in the community of the *R. etli* population with succinate chosen as C-source at a total uptake rate of 4.16 mmol/gDWC/h (consistent with values used for single growing *R. etli*).

Fig 4 shows the competence of members for succinate, where N-fixing member consumed a smaller share of succinate (27.2%) compared to the growing member (72.8%), indicating an effective division of labor where the non-growing focuses on N-fixation supporting the growing member via ammonium transfer. Molecular nitrogen (N₂), set as the sole N-source to this community, was fixed to NH₃ by non-growing cells and this was further consumed by the growing member. Of the total uptake of oxygen, 48% was used in non-growing cells allowing ATP generation required for N-fixation while the rest was used for respiration of growing cells. Results showed a total community growth rate of 0.1933 h ⁻ ¹ (µ_1 _+ µ_2_) which was lower than that observed on ammonium (NH₃) as N-source. This reduction can be attributed to the energy-intensive process of N-fixation, where ATP demand is high for breaking the triple bond of N₂. Accordingly, lower nitrogen uptake (1.469 mmol/gDWC/h) and higher CO_2_ emission (9.517 mmol/gDWC/h) rates were obtained in community compared to the case with ammonia as N-source.

Computational results on PHB content of N-fixing cell (see Fig 4) showed a value of 50%, while it was 16% for the growing member, as formulated. Furthermore, the simulated value of µ1^*^ matched the definition provided in Eq. 6, which accounts for cell differentiation into the non-growing N-fixing state. These results indicated that the computational results reflected the assumptions and formulations used in modeling.

Considering identical C-source (succinate) uptake rate, results showed the superiority of ammonia among the examined N-sources (NH_3,_ NO_2_, NO_3_ and N_2_), followed by nitrite and then molecular nitrogen with regards to growth rates of *R. etli*. However, molecular nitrogen (N₂) led to a slightly higher growth rate as opposed to nitrate (NO₃). This is due to the difference in energy requirements of NO₃ assimilation and N₂ fixation. Nitrate reduction consumes reducing power equivalent to 12 moles of ATP per mole of ammonium produced, whereas N₂ fixation requires only 8 moles of ATP per mole of ammonia produced.

### Robustness analysis on exchange fluxes between two members in community

In our community modeling approach, the exchange of metabolites between N-fixing and growing cells was restricted only to ammonia. However, the symbiotic metabolic models of N-fixing *R. etli* account for other exchanges such as amino acids alongside NH_3_ between the host and N-fixing *R. etli*. These exchanges have been experimentally proven to enhance plant growth [20–23]. It should be noted that in our population, both members have the same metabolism and grow on the same C-source, so exchanging amino acids would not have the same effect as observed in the symbiosis between *R. etli* and plant roots. Nevertheless, the impact of these exchanges was explored to provide a comprehensive understanding of behavior of this community.

To explore the effect of exchanging other metabolites containing both C- and N-atoms on total community growth, exchanges of some amino acids were allowed between members (in addition to ammonia) one at a time. The exchange of each amino acid (including alanine, aspartate, glutamate, and glutamine), with succinate uptake fixed at 4.16 mmol/gDWC/h, did not alter the community growth rate, although a new flux distribution was achieved. This indicates the existence of multiple optimal solutions for the underlying linear programming problem.

**Fig 5:**
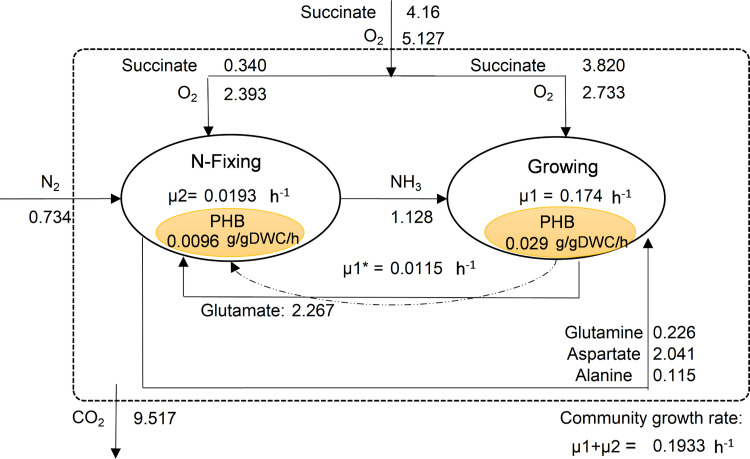
Flux distribution of one of optimal solutions in N₂ fixing community of R. etli with allowed exchange of NH_3_, Glutamine, Glutamate, Aspartate, and Alanine. Numbers shown next to arrows show flux values in mmol/gDWC/h.

Then all the exchanges were allowed simultaneously and the flux distribution of one of optimal solutions is presented in Fig 5. Results indicated that the community growth rate remained unchanged when other metabolites alongside ammonia were exchanged between members. Interestingly, this result implies that linear programming optimization of the community model yields multiple optimal solutions, as the same growth rate was achieved despite differences in flux distributions within the community consistent with the fact that FBA returns a single optimal solution, even though alternative solutions with identical objective values but different flux profiles may also exist.

The distinction between free-living N-fixing *R. etli* community and its symbiotic form, where metabolite exchange is beneficial, lies in the differing objectives of these two systems and the variation in their substrates. In free-living form, both members consume the same substrate (e.g., succinate) and exchange only ammonia. In contrast, during symbiosis, the plant provides the bacteria with a carbon source, while the bacteria supply the plant with amino acids and fixed nitrogen in the form of ammonia. As a result, metabolite exchange in the symbiotic state enhances the plant’s growth rate and is essential for the function of *R. etli*. However, in free-living form, exchanging metabolites other than ammonia offers no advantage, as the shared substrate and similar metabolic objectives of the two members eliminate the need for additional exchanges.

**Fig 6:**
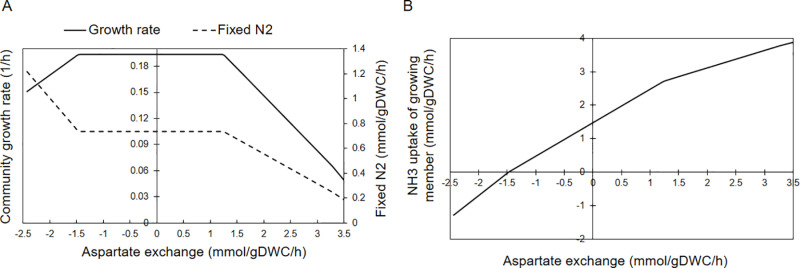
Effect of aspartate exchange between the growing and non-growing members of the R. etli community on (A) community growth rate ( µ₁ + µ₂) and N₂ fixation, and **(B)** **NH₃** **uptake by the growing cell.** Negative aspartate fluxes indicate transport from the non-growing to growing member, while positive values indicate the reverse. Also, negative NH₃ fluxes represent its export by the growing member to outer space of community, whereas positive values denote NH₃ uptake by the growing from non-growing member.

To investigate the population behavior under conditions where metabolite exchange would occur at specific values due to environmental or metabolic shocks, robustness analysis was performed by one at a time exchange allowance of some amino acids (aspartate, alanine, glutamate, and glutamine) alongside NH_3_ at succinate uptake fixed at 4.16 mmol/gDWC/h. Consistent trends of rates of community growth, N-fixation and exchange of ammonia to growing cells were observed for the allowed amino acids and hence only the results for aspartate exchange are presented in Fig 6 as an example, while results of other amino acids are given in supplementary S3 File.

As can be seen in Fig 6A, at low rates of amino acid exchange, the growth rate remained unaffected, achieving the same optimal growth rate as in the case of no exchange. However, higher exchange rates in any direction led to decreased growth rate. Exchange of amino acid (aspartate in Fig 6) from the non-growing member to growing member resulted in increased N-fixation providing excess amino acid to the growing member leading to NH3 transport to the extracellular space (the negative fluxes of NH3 in Fig 6B). Simultaneously, the excess carbon received by growing member, exited the community as CO_2_ (S4 file). At high rates of aspartate exchange from the growing to non-growing member (see Fig 6A), N₂ fixation decreased due to non-growing member metabolizing the exchanged aspartate leading to formation of NH₃ for transfer to growing member, thereby reducing its reliance on N₂ fixation. Since aspartate synthesis by growing member needed more NH_3_, Fig 6B shows that NH₃ uptake of growing member increased. In terms of substrate consumption rate, the portion of substrate uptake by each member shifts based on the direction of exchange. As the member supplying the exchanged metabolite consumes a larger share of the available resources.

### Sensitivity analysis on member abundance in community-like population

**Fig 7:**
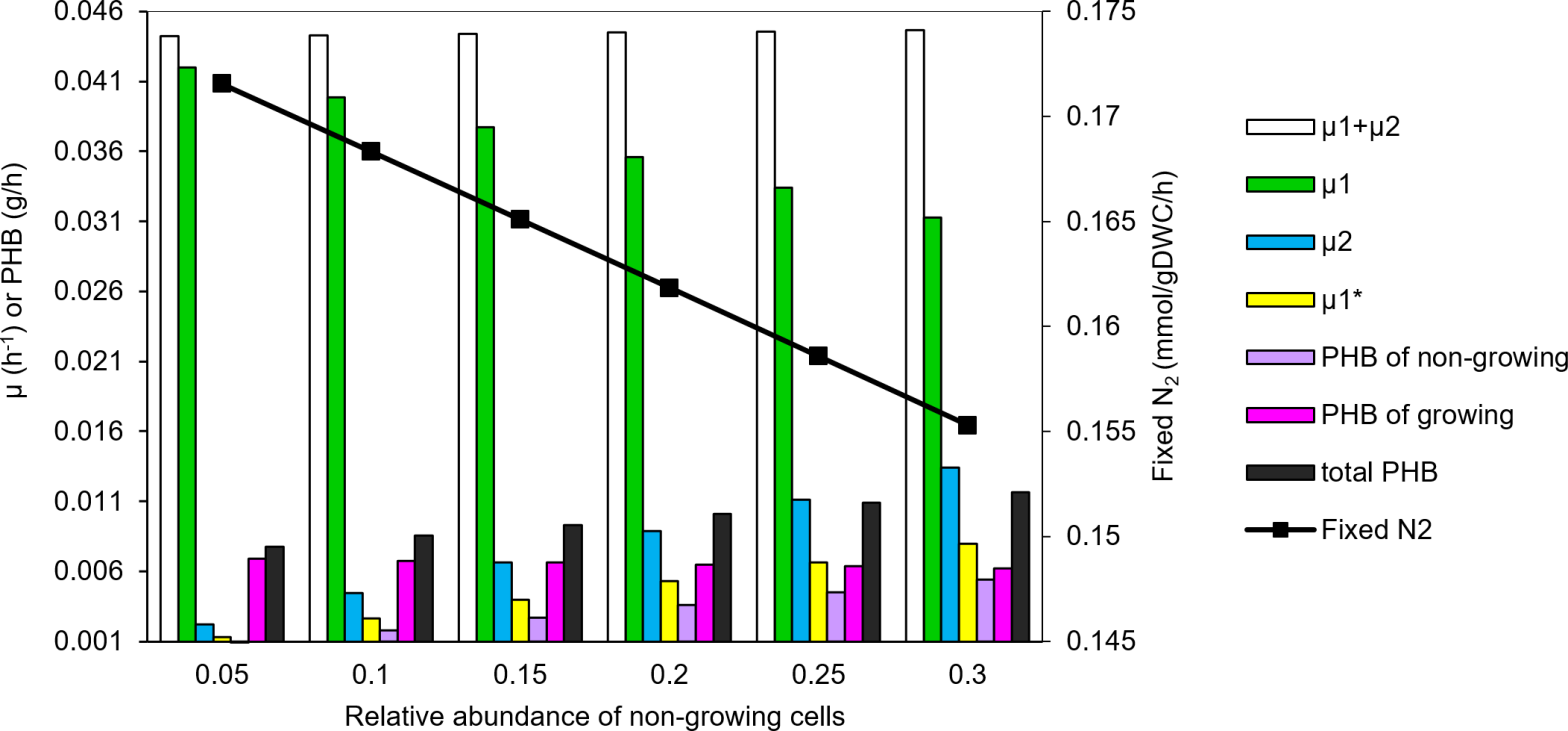
Effect of abundance of N-fixing cells on community behavior. µ denotes growth rate, 1 and 2 refer to growing and N-fixing cells, respectively, and * shows differentiation.

In the previous section, an abundance of 10% was assumed for N-fixing non-growing cells to simulate the behavior of N-fixing community of *R. etli*. To investigate the effect of variation of abundance of members on community behavior, a range of 5–30% of the total population was examined for non-growing cells at fixed succinate uptake of 4.16 mmol/gDWC/h. Selection of this range was based on the reported frequency of heterocyst part of filamentous N-fixing cyanobacteria [28] as no specific data was available for *R. etli*. For each abundance level, the constant coefficient in Eq. 6 (initially $\frac{1}{9}$) was recalculated to ensure that the relative abundances remain constant over time. This led to new coefficients of $\frac{1}{19},\;\;\frac{1}{9},\;\;\frac{3}{17},\;\;\frac{1}{4},\;\;\frac{1}{3}\;and\;\;\frac{3}{7}$ for non-growing member abundances of 5, 10, 15, 20, 25, and 30% of the total population, respectively. The effect of abundance of N-fixing cells on contribution of each member in community growth rate as well as fluxes of fixed N_2_ and PHB formation are illustrated in Fig 7.

As can be seen in Fig 7, growth rate of the growing member (µ_1_) showed 25% decrease with increase of non-growing abundance from 5 to 30%. This decline is due to the fact that a larger portion of the available substrate is consumed by non-growing cells, leaving less substrate for growing member. Despite this decrease, the overall growth rate of the community (µ_1_ + µ_2_) remains relatively constant. This is attributed to an increase in the differentiated cells (µ_1_^*^) transitioning into non-growing cells to maintain the constant relative abundances. Additionally, PHB production by non-growing member increased with its relative abundance. This is directly related to the rise in µ_1_^*^, as the constraint of Eq. 4 relates PHB production to µ_1_^*^. The sum of µ_1_^*^ and PHB produced by non-growing member constitutes µ_2_ and subsequently µ_2_ increased with non-growing member abundance. Additionally, the PHB content of the growing member slightly decreased with an increase in the abundance of N-fixing cells, likely due to a reduction in µ₁. In contrast, the total community PHB, calculated as the sum of PHB from both non-growing and growing cells, increased with a higher abundance of non-growing cells. This indicates that, because non-growing cells have a larger PHB content, their increased abundance raises the overall PHB level in the community.

Another significant change in population behavior with increasing non-growing member abundance (from 5 to 30%) is the 10% reduction of N-fixation. Despite the higher substrate consumption, the increased PHB production, regulated by the constraint linking PHB production to the differentiation rate of cells into non-growing members, results in less available energy for N-fixation. Consequently, the model suggests that a lower abundance of non-growing members benefits the overall population, both in terms of growth rate and enhanced N-fixation.

### Cytochrome *bd* oxidase activation at high levels of oxygen consumption

As discussed in introduction section, nitrogenase, the key enzyme involved in N-fixation, is highly sensitive to elevated oxygen levels. One of the strategies employed by N-fixing organisms to mitigate this challenge is the presence of two distinct cytochrome oxidases in their oxidative phosphorylation pathways. Cytochrome *bd*, in particular, demonstrates a higher rate of oxygen consumption compared to cytochrome *c* for the same amount of ATP generation. This allows cytochrome *bd* to become active under high oxygen conditions, generating ATP while simultaneously scavenging excessive oxygen to protect nitrogenase.

Although the cytochrome *bd* reaction has been incorporated into some *Rhizobium* metabolic models, its activation under high oxygen conditions has not been previously investigated. Here, the activation of the cytochrome *bd* reaction under elevated oxygen levels was examined in our model. Simulations were conducted using the N-fixing community model with a fixed succinate uptake rate of 4.16 mmol/gDWC/h and members abundances of 1:9 for non-growing and growing member, respectively. The effect of increasing oxygen uptake on growth rate and flux through cytochrome oxidases was assessed.

**Fig 8:**
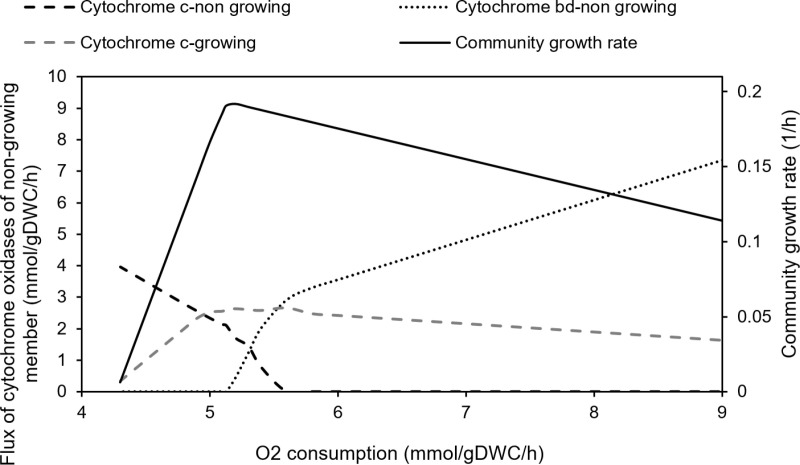
Effect of high oxygen uptake rate on the activation of cytochrome bd for scavenging excess oxygen and its impact on the growth rate, simulated by the R. etli model at fixed succinate uptake of 4.16 mmol/gDWC/h.

As shown in Fig 8, the model became infeasible at community oxygen uptake of lower than 4.3 mmol/gDWC/h. This represents the minimum oxygen required to metabolize 4.16 mmol/gDWC/h of succinate. As oxygen uptake increased, the growth rate rose, and oxygen consumption was through cytochrome *c* oxidase in both growing and non-growing members. However, with increase in oxygen, the fraction of oxygen consumed by the non-growing member decreased, while that of growing member increased, consistent with the higher growth rate. At 5.2 mmol/gDWC/h of oxygen, growth rate reached its maximum, with all oxygen uptake in the N-fixing member being directed through its cytochrome *c*, generating more ATP and supporting maximum growth. Up to this point, when oxygen was insufficient to fully metabolize succinate into biomass precursors, formate was produced and excreted into the extracellular space.

By increasing oxygen uptake rate beyond 5.2 mmol/gDWC/h, up to approximately 5.5 mmol/gDWC/h, both cytochrome *c* and cytochrome *bd* oxidases in the N-fixing member became active. The contribution of cytochrome *bd* increased with rising oxygen. Beyond 5.5 mmol/gDWC/h, only cytochrome *bd* remained active in the N-fixing member, enabling the cell to scavenge excess oxygen and by every 1 mmol/gDWC/h increase in O_2_ consumption growth rate dropped by 12%. However, the growth rate declined at higher oxygen uptake rates due to the lower ATP yield of cytochrome *bd*, which was insufficient to sustain maximum growth. Additionally, as the growth rate decreased, excess carbon left the system as CO₂ in the presence of high oxygen levels.

These results suggest a metabolic adjustment to balance energy generation and oxygen detoxification under varying oxygen conditions.

### Principal component analysis on flux distribution of *R. etli* with various N-sources

As previously discussed, the type of N-source significantly influences *R. etli* growth and carbon usage efficiency. To compare flux distributions and identify differences across four N-sources (NH₃, NO₂, NO₃, and N₂), principal component analysis (PCA), a powerful tool for comparing observations of the same variables under varying conditions, was employed. PCA has been utilized to compare metabolic flux distributions obtained under different conditions [38,39]. This method provides a systematic approach to reducing the complexity of data by transforming flux distributions into principal components, enabling the identification of key differences and patterns across conditions. By focusing on principal components that explain the highest variance, PCA effectively highlights the metabolic reactions contributing most to the observed differences, offering valuable insights into condition-dependent flux variations. By applying PCA, it becomes possible to identify the critical reactions responsible for distinguishing the flux distributions under the four nitrogen source conditions.

**Fig 9:**
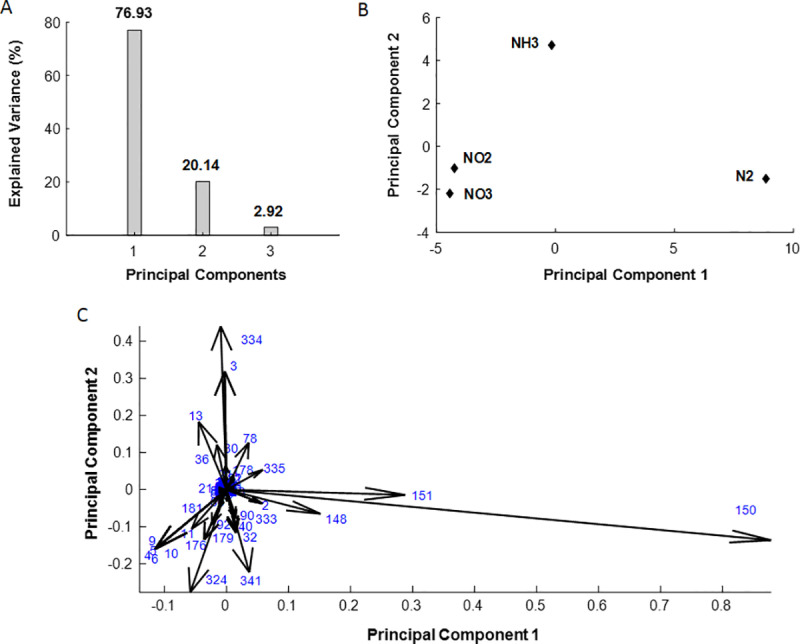
Results of PCA comparing flux distributions of R. etli under various N-sources. A: Explained variance diagram, B: Score plot, and C: Loading plot from PCA results comparing different nitrogen source substrates. Numbers in loading plot indicate reaction numbers as presented in supplementary S1 file.

In this study, however, separate models were used for community like population of *R. etli* capable of N-fixing with members abundances of 1:9 for non-growing and growing member, respectively, and the growing cells on NH₃, NO₂, and NO₃, at fixed succinate uptake rate of 4.16 mmol/gDWC/h making a direct comparison challenging as a result of the dissimilar size of the flux vectors. To address this issue, the fluxes from both the growing and non-growing members in the community model were summed together. This approach ensured that the size of the flux vectors across all four cases were consistent, enabling a meaningful comparison using PCA. The results of PCA are shown in Fig 9.

Fig 9A illustrates the explained variance plot for the four examined cases. It shows the contribution of the principal components (PCs) to the total variability in the data. The first principal component (PC1) accounts for approximately 77% of the variance, indicating that it captures the majority of the variability in the dataset. The second principal component (PC2) explains around 20% of the variance, showing that while it contributes to the data variability, its significance is much smaller compared to PC1. Together, PC1 and PC2 explain majority of the variance, demonstrating that the data’s complexity can be effectively captured and analyzed within a two-dimensional space.

Fig 9B illustrating the PCA score plot clearly demonstrates the differences between the flux distributions related to the four N-sources. This plot provides a visualization of the separation between the four N-sources (N₂, NH₃, NO_2_ and NO_3_) in the reduced principal component (PC) space. The distribution of points reveals that the fluxes associated with N₂ are significantly distinct from those of the other N-sources, as evidenced by the large separation along the first principal component (PC1). This suggests that the metabolic network undergoes substantial reorganization when N₂ is utilized, likely due to the high energy demands of N-fixation. Conversely, the flux distributions for NO₂ and NO₃ are closely positioned, reflecting a significant similarity in their metabolic profiles. This similarity arises from the fact that the metabolic pathways for NO₃ and NO₂ utilization share nearly identical reaction networks, with the only difference being a single reaction in biochemical reaction network where NO₃ is reduced to NO₂. Beyond this step, the subsequent metabolic pathways are entirely identical, resulting in minimal variation in their flux distributions.

The separation of NH₃ from other nitrogen sources along the second principal component (PC2) highlights the unique metabolic flux distribution associated with this N-source. This arises from the fact that ammonia can be directly utilized to synthesize biomass components, such as amino acid and nucleotide, thereby reducing the energy requirement typically needed for nitrogen fixation. The distinct clustering of N₂ further emphasizes the metabolic burden associated with N-fixation, including increased ATP demand. The clear separation of the points in the score plot suggests that the N-source significantly impacts the metabolic behavior of the system, with distinct metabolic states emerging under each condition.

Fig 9C shows the loading plot resulting from the PCA for key metabolic reactions. This plot allows us to assess the relative importance of each reaction in distinguishing between the studied N-sources. The position of each reaction in the plot indicates the extent of its contribution to the first principal component (PC1) and the second principal component (PC2). Reactions that are farther from the center of each axis have a greater influence on that component. For instance, reaction such as 150 on the right side of the plot with larger lengths, significantly contribute to PC1, while a reaction like 334 at the top of the plot with a greater length, shows a stronger influence on PC2.

Analysis of the loading plot revealed that the reactions with the highest contributions to PC1 and PC2 could be classified into three main metabolic pathways: oxidative phosphorylation, tricarboxylic acid (TCA) cycle, and glycolysis. Among these, oxidative phosphorylation plays a pivotal role, as reaction 150 (corresponding to ATP synthesis) exhibited the greatest distance along PC1. This highlights its importance in differentiating flux patterns, likely due to the varying energy demands across cases. For instance, N_-_fixation, which relies on the energy-intensive nitrogenase enzyme, significantly increases ATP demand, influencing the flux distribution in this pathway.

The TCA cycle also emerged as a critical contributor, with key reactions such as the conversion of malate to oxaloacetate (OAA), succinate to succinyl-CoA (sucCoA), and OAA to citrate showing strong influences on the principal components. This reflects the central role of the TCA cycle in producing energy, reducing equivalents (NADH and FADH_2_), and precursor metabolites. The shifts in fluxes through these reactions align with the metabolic adaptations required to balance the energy and reducing power supply at different N-sources. Previous studies also emphasized the importance of TCA cycle on N-fixation in *Rhizobium* [40,41]. Additionally, glycolytic reactions, including the conversion of glucose-6-phosphate (G6P) to fructose-6-phosphate (F6P), phosphoenolpyruvate (PEP) to pyruvate, and pyruvate to acetyl-CoA (AcCoA), also contribute significantly to the observed variability. These reactions are integral to providing both energy and carbon intermediates, particularly under N-fixing conditions where the energetic and biosynthetic demands are high. The conversion of malate to pyruvate also suggests anaplerotic flux adjustments to sustain the metabolic needs of N-fixation and assimilation. Together, these findings underscore the dynamic reorganization of metabolic fluxes in response to N-source availability, with oxidative phosphorylation, TCA cycle, and glycolysis serving as major pathways of adaptation.

## Conclusions

This study presents a comprehensive computational investigation of *R. etli* metabolism in its free-living state, addressing a significant gap in current models that predominantly focus on symbiotic state. By refining the existing *i*OR363 model through the incorporation of a biomass formation reaction and subsequent curation, the growth of free-living *R. etli* was accurately simulated using various nitrogen sources, with ammonia yielding the highest growth rate (0.259 h ⁻ ¹) at a fixed succinate uptake rate of 4.16 mmol/gDWC/h.

Furthermore, a novel two-member, community-like metabolic model was developed to capture the inherent heterogeneity of free-living population, where a subset of cells differentiated into a non-growing nitrogen-fixing state. Using the XFBA approach to maintain fixed cell abundances, simulations revealed that the overall community growth rate decreased to 0.1933 h ⁻ ¹ under nitrogen fixation due to the high energy demand associated with N₂ assimilation. Sensitivity analyses further demonstrated that increasing the proportion of nitrogen-fixing cells adversely affects the growth of growing member, underscoring the importance of specific cellular distribution for optimal performance.

With regard to metabolite exchange experiments, except for ammonia, the exchange of other amino acids does not enhance community growth and may even reduce it under excessive exchange conditions. This finding highlights a key distinction between the free-living and symbiotic states: in free-living, amino acid exchange does not increase the optimal growth, while the symbiotic community depends critically on metabolite exchange to grow. This observation suggests that multiple optimal solutions existence, as the same growth rate can be maintained despite differences in flux distributions within the community. Principal Component Analysis (PCA) identified key metabolic pathways of oxidative phosphorylation, TCA cycle, and glycolysis as critical contributors to flux variations across different N-sources. Finally, the activation of cytochrome *bd* oxidase under high oxygen uptake was shown to protect nitrogenase by scavenging excess oxygen, though at the expense of reduced ATP generation and hence growth rate.

Overall, these findings provide valuable insights into the free-living metabolism of *R. etli* and offer a robust platform for further research and metabolic engineering of diazotrophic bacteria.

## Supporting information

S1 file Curated model of growing member. (XLSX)

S2 file Community model of free-living *R. etli.* (XLSX)

S3 file Results of robustness analysis on exchange fluxes between two members in community (for glutamate, glutamine, and alanine). (XLSX)

S4 file Results of amino acids exchange experiments. (DOCX)

S5 file Raw data underlying all figures and tables in the manuscript. Includes numerical values used to generate plots, flux distributions, and other results.
(XLSX)
