# Supplementary material for: Diazotrophic growth of free-living Rhizobium etli: Community-like metabolic modeling of growing and non-growing nitrogen-fixing cells
Source: PLoS One. 2025 Jun 27;20(6):e0325888. doi: 10.1371/journal.pone.0325888 (PMC12204555; doi:10.1371/journal.pone.0325888)
Supplement: S4 file — (DOCX) [file pone.0325888.s004.docx]

Figure S1. Effect of glutamine exchange between the growing and non-growing members of the R. etli community on the community growth rate (µ1 + µ2), N_2_ fixation and NH_3_ uptake of growing member. Negative fluxes of glutamine indicate transport from the non-growing to the growing member, while positive fluxes indicate transport from the growing to the non-growing member. Negative fluxes of NH_3_ indicate its export to the outer space of community by growing member

Figure S2. Effect of glutamate exchange between the growing and non-growing members of the R. etli community on the community growth rate (µ1 + µ2), N_2_ fixation and NH_3_ uptake of growing member. Negative fluxes of glutamate indicate transport from the non-growing to the growing member, while positive fluxes indicate transport from the growing to the non-growing member. Negative fluxes of NH_3_ indicate its export to the outer space of community by growing member

Figure S3. Effect of alanine exchange between the growing and non-growing members of the R. etli community on the community growth rate (µ1 + µ2), N_2_ fixation and NH_3_ uptake of growing member. Negative fluxes of alanine indicate transport from the non-growing to the growing member, while positive fluxes indicate transport from the growing to the non-growing member. Negative fluxes of NH_3_ indicate its export to the outer space of community by growing member
